# Supplementary material for: Monte Carlo Models for Sub-Chronic Repeated-Dose Toxicity: Systemic and Organ-Specific Toxicity
Source: Int J Mol Sci. 2022 Jun 14;23(12):6615. doi: 10.3390/ijms23126615 (PMC9224506; doi:10.3390/ijms23126615)
Supplement: Supplementary file 1 [file ijms-23-06615-s001.zip › Supplementary figures and tables S25-28.pdf]

Monte Carlo Models for Sub-Chronic Repeated-Dose Toxicity: Systemic and Organ-Specific Toxicity

Gianluca Selvestrel [1,†,*] and Giovanna J. Lavado [1,†,*], Alla P. Toropova [1], Andrey A. Toropov [1], Domenico Gadaleta [1], Marco Marzo [1], Diego Baderna [1] and Emilio Benfenati [1]

[1] Laboratory of Chemistry and Environmental Toxicology, Department of Environmental Health Sciences, Istituto di Ricerche Farmacologiche Mario Negri IRCCS, via Mario Negri 2, 20156 Milan, Italy

* Correspondence: gianluca.selvestrel@marionegri.it (G.S.); giovanna.lavado@marionegri.it (G.J.L.)

† These authors contributed equally to the work

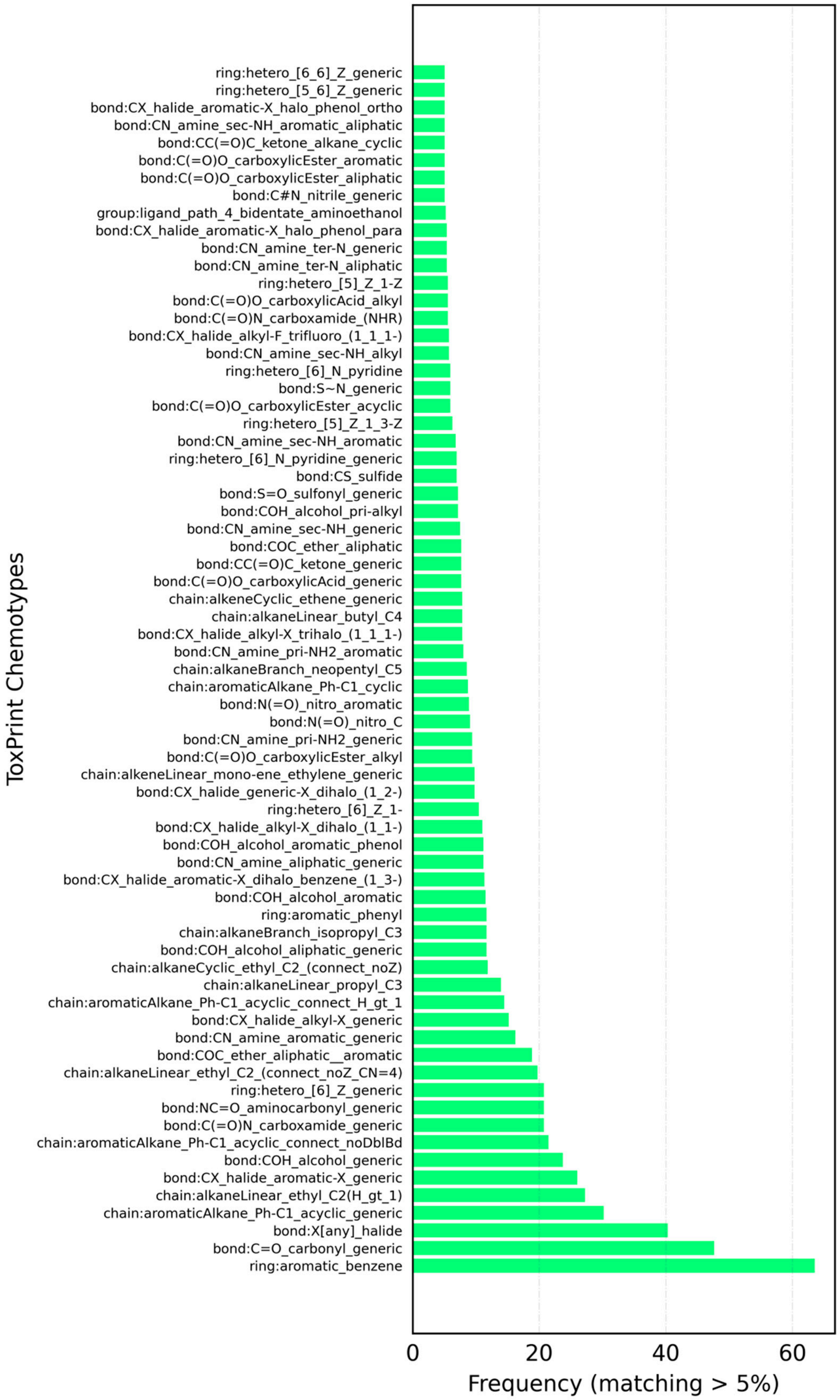

Figure S1. Histogram of ToxPrint Chemotypes identified in the general dataset.

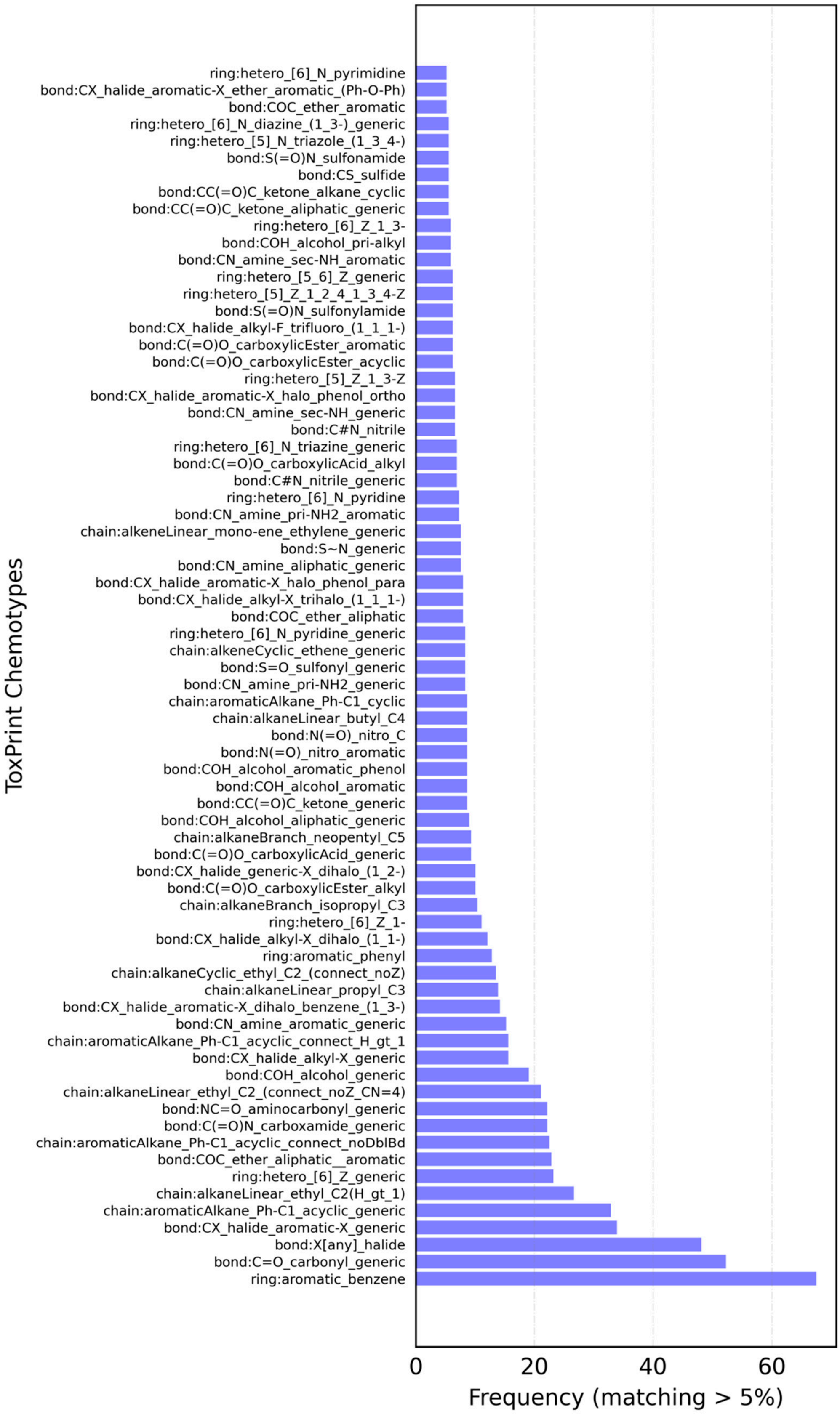

Figure S2. Histogram of ToxPrint Chemotypes identified in the kidney dataset.

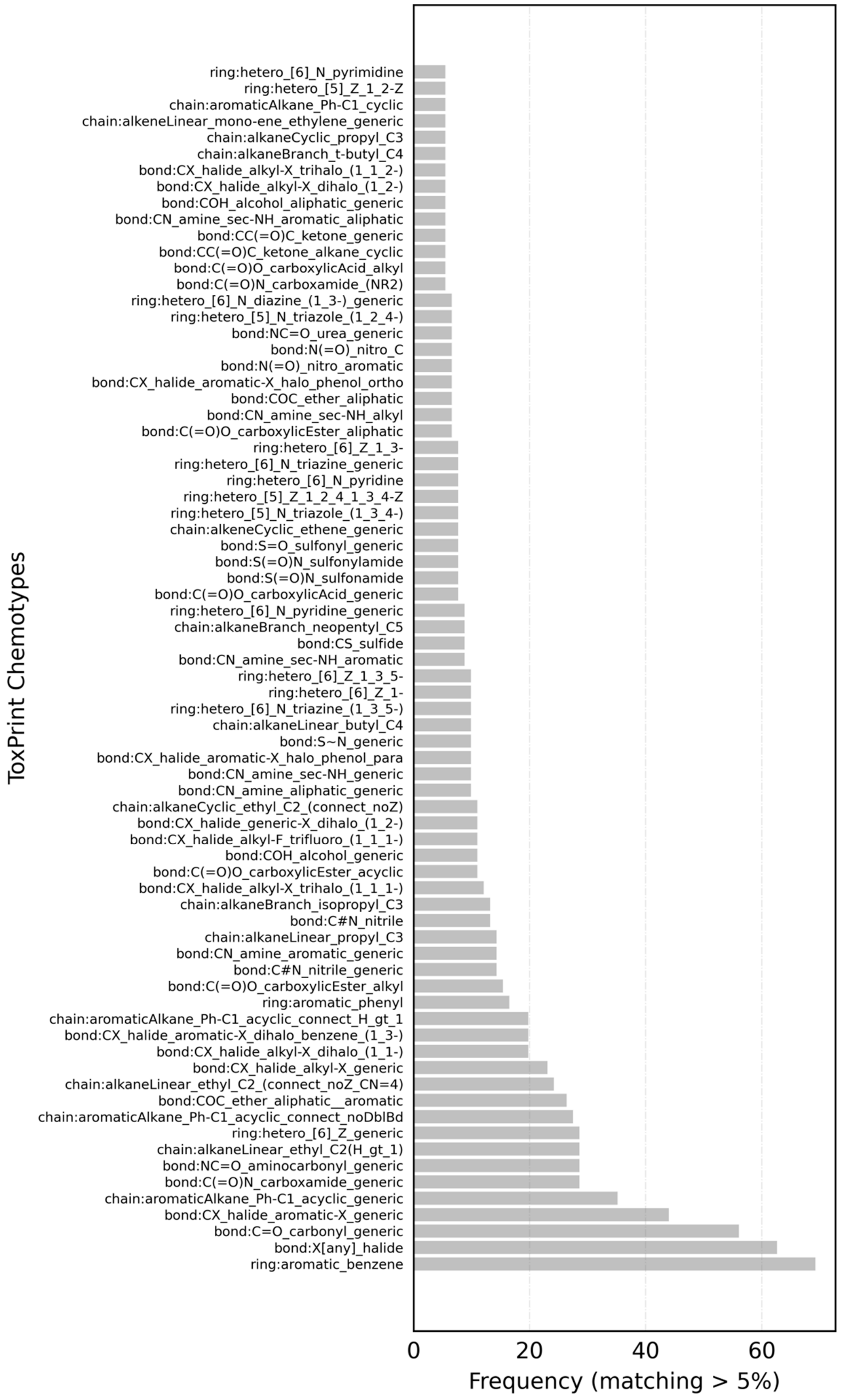

Figure S3. Histogram of ToxPrint Chemotypes identified in the brain dataset.

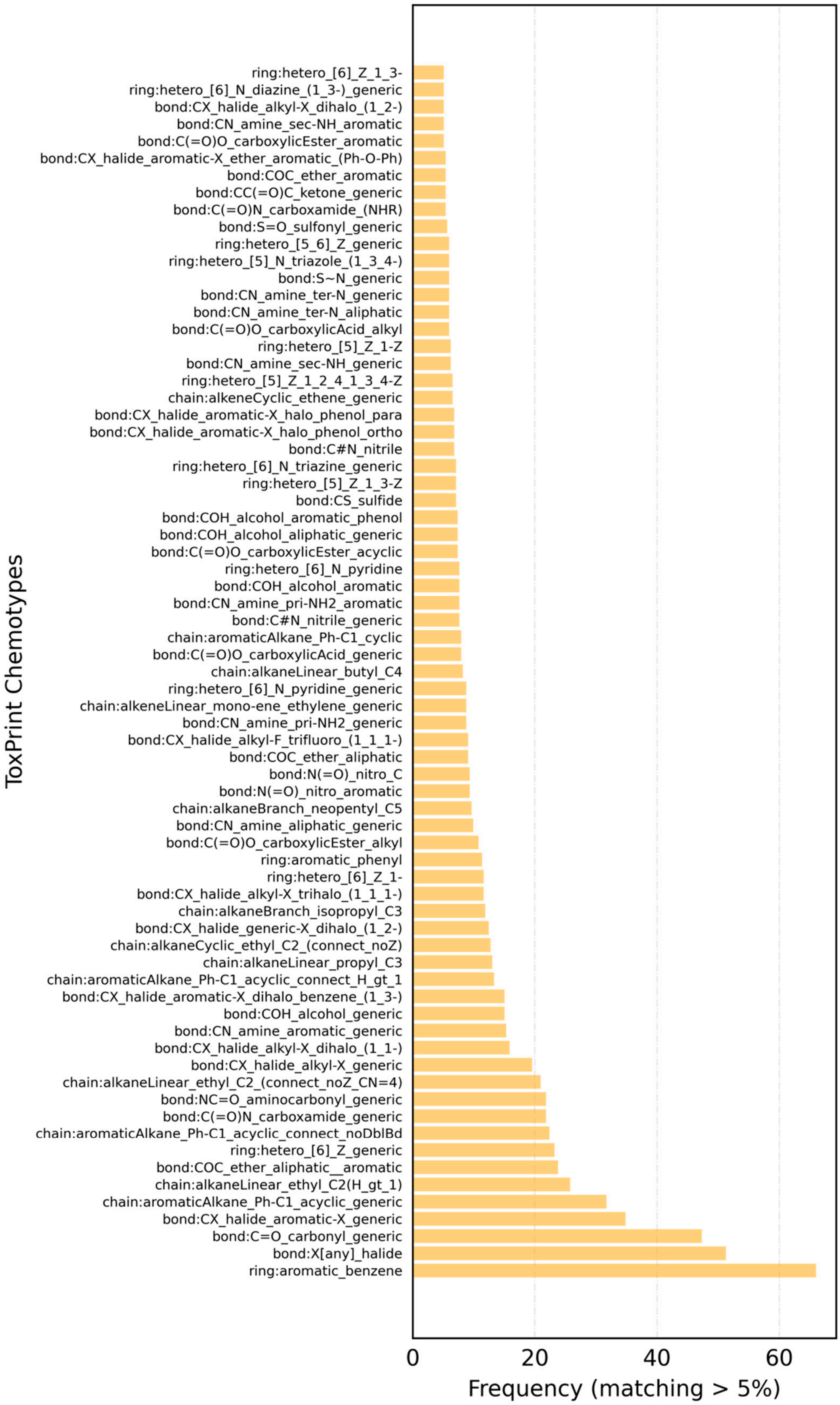

Figure S4. Histogram of ToxPrint Chemotypes identified in the liver dataset.

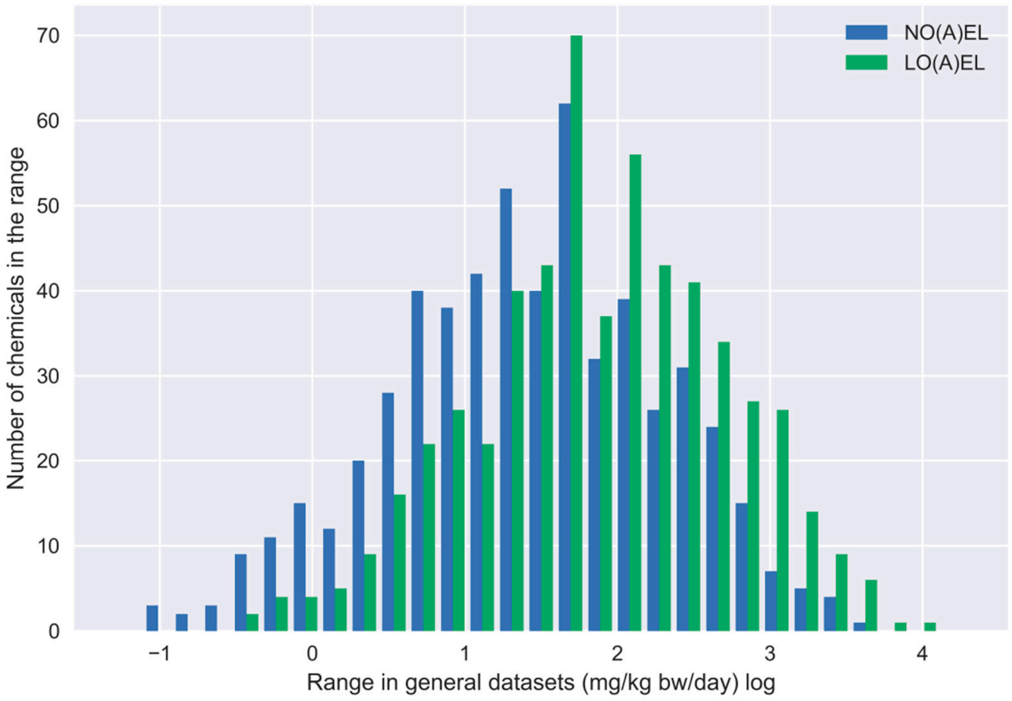

Figure S5. Frequency distribution of the NOAEL and LOAEL values in the general dataset.

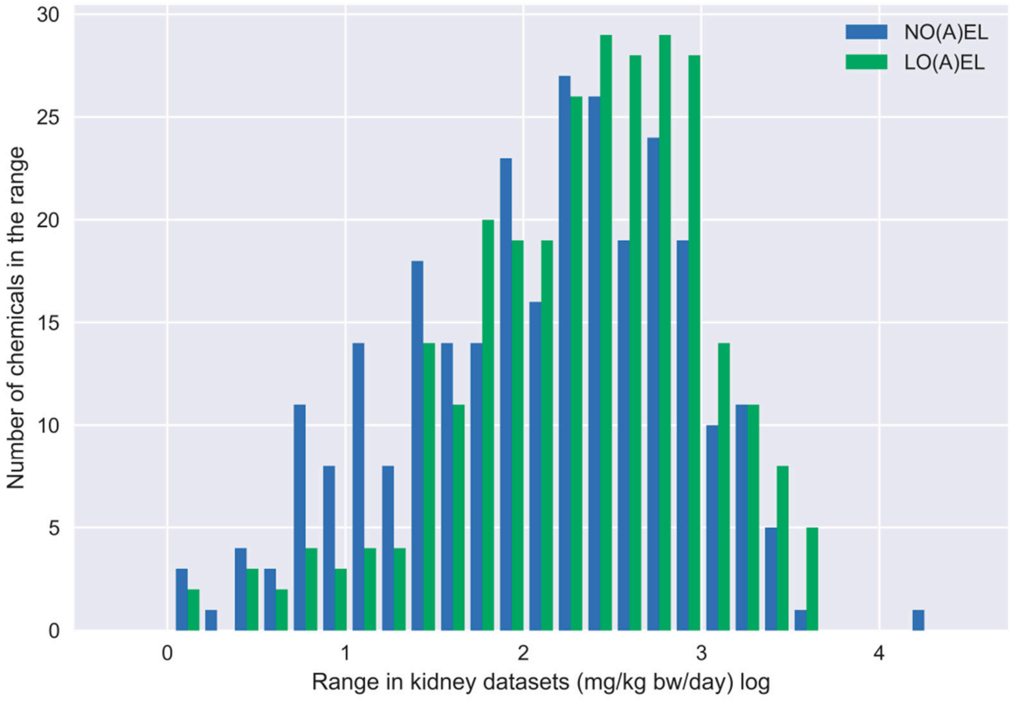

Figure S6. Frequency distribution of the NOAEL and LOAEL values in the kidney dataset.

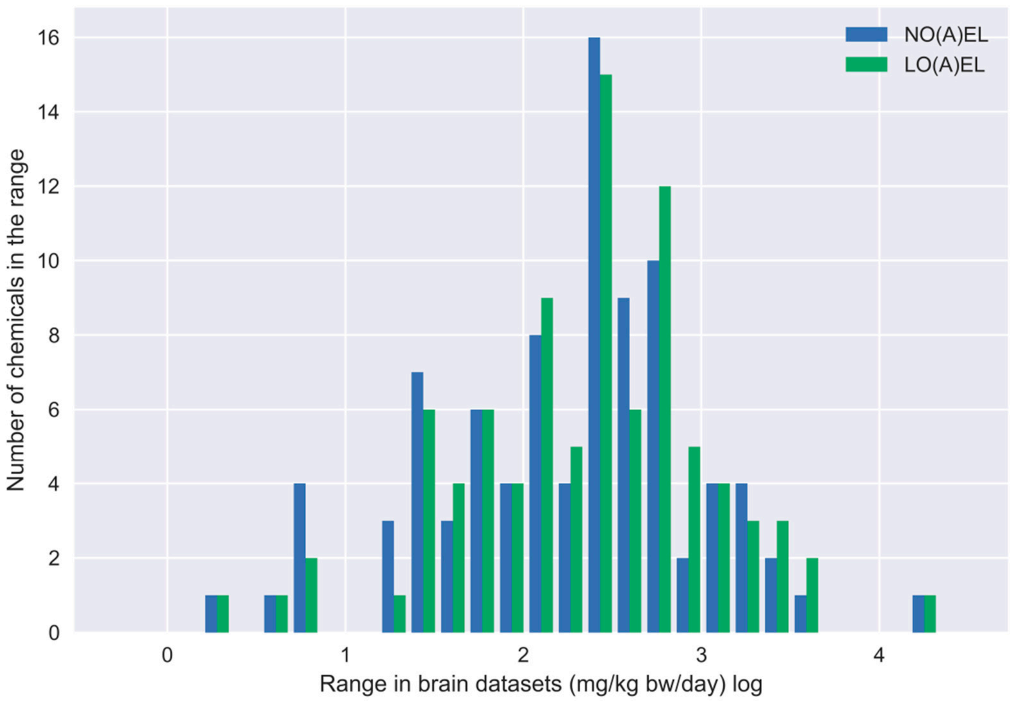

Figure S7. Frequency distribution of the NOAEL and LOAEL values in the brain dataset.

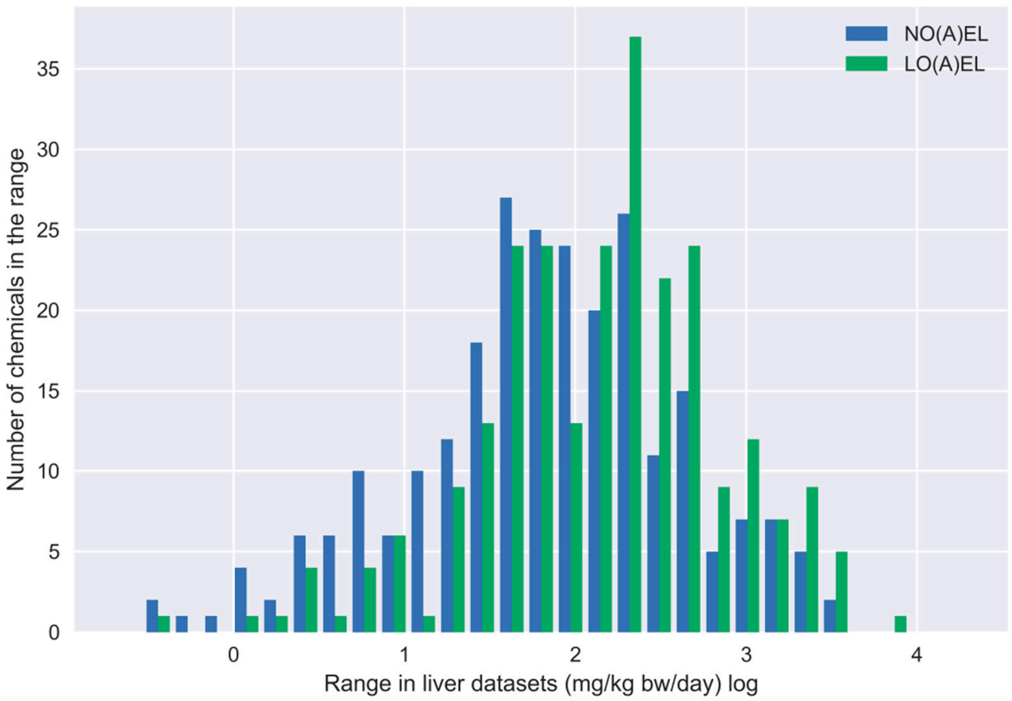

Figure S8. Frequency distribution of the NOAEL and LOAEL values in the liver dataset.

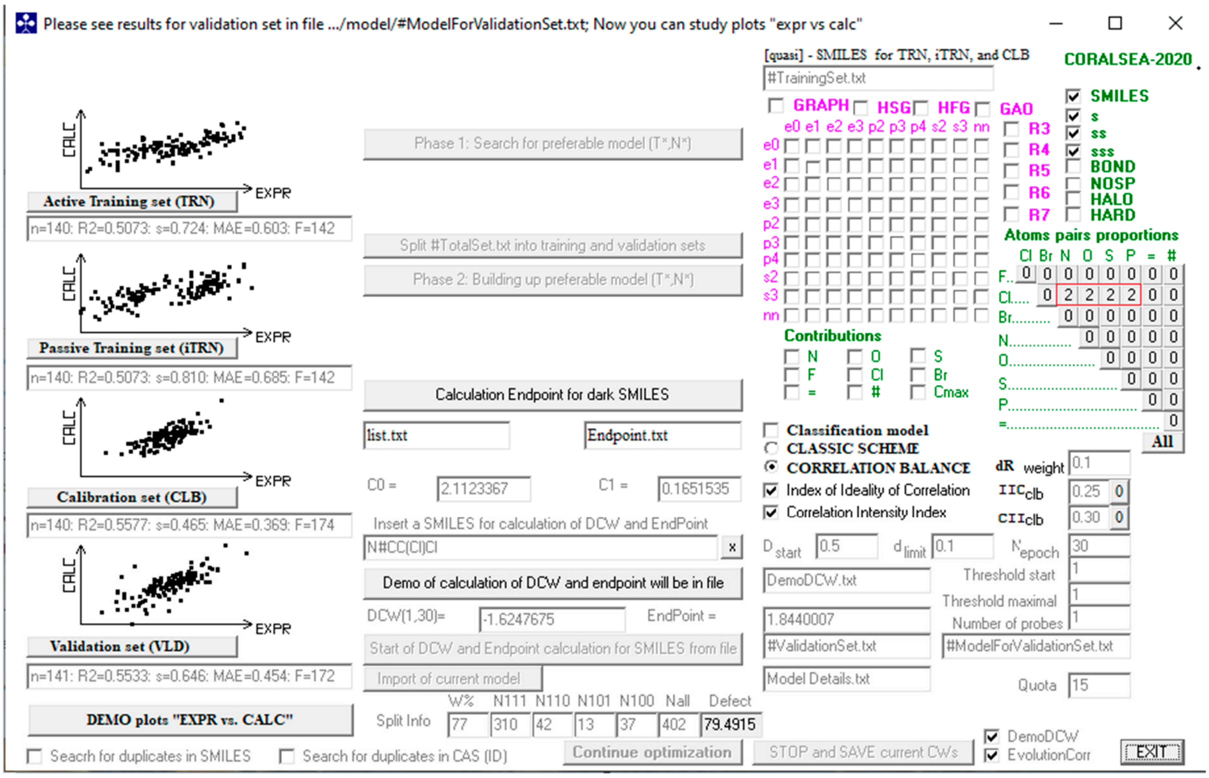

Figure S9. Screenshot of the method M1 utilized to build up the general NOAEL model in the CORAL software.

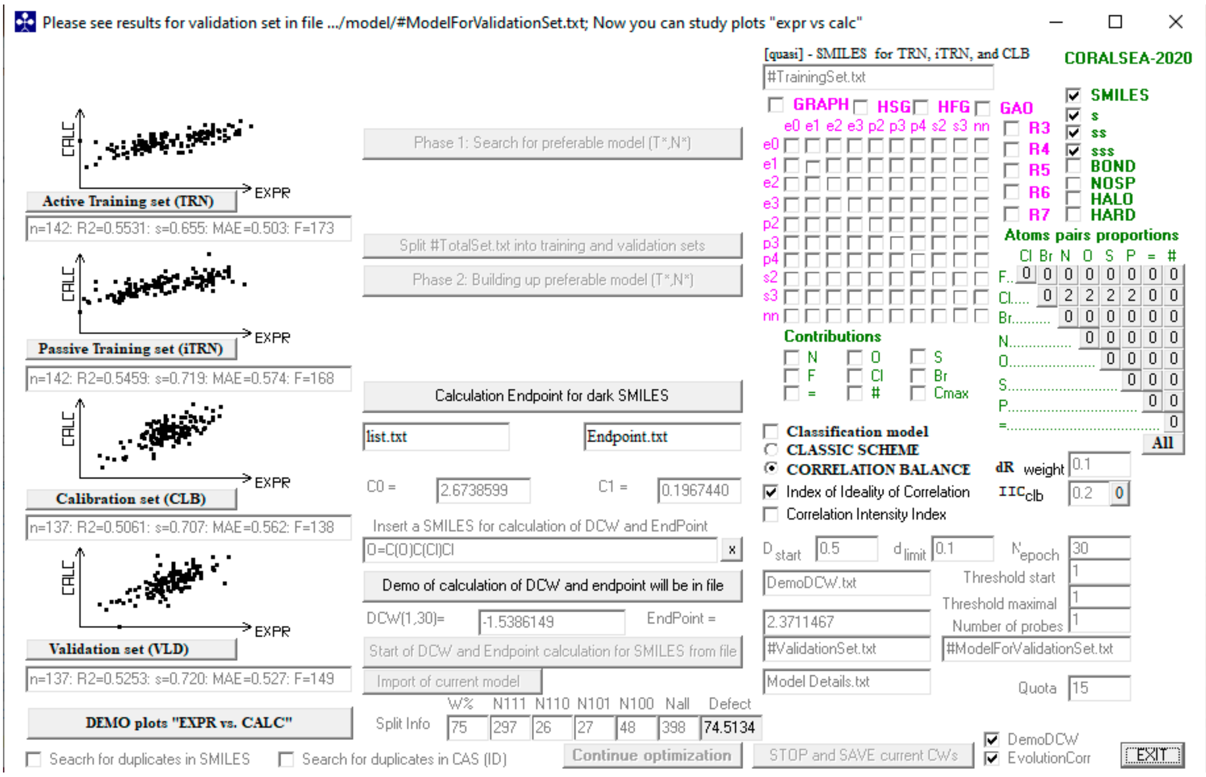

Figure S10. Screenshot of the method M2 utilized to build up the general LOAEL model in the CORAL software

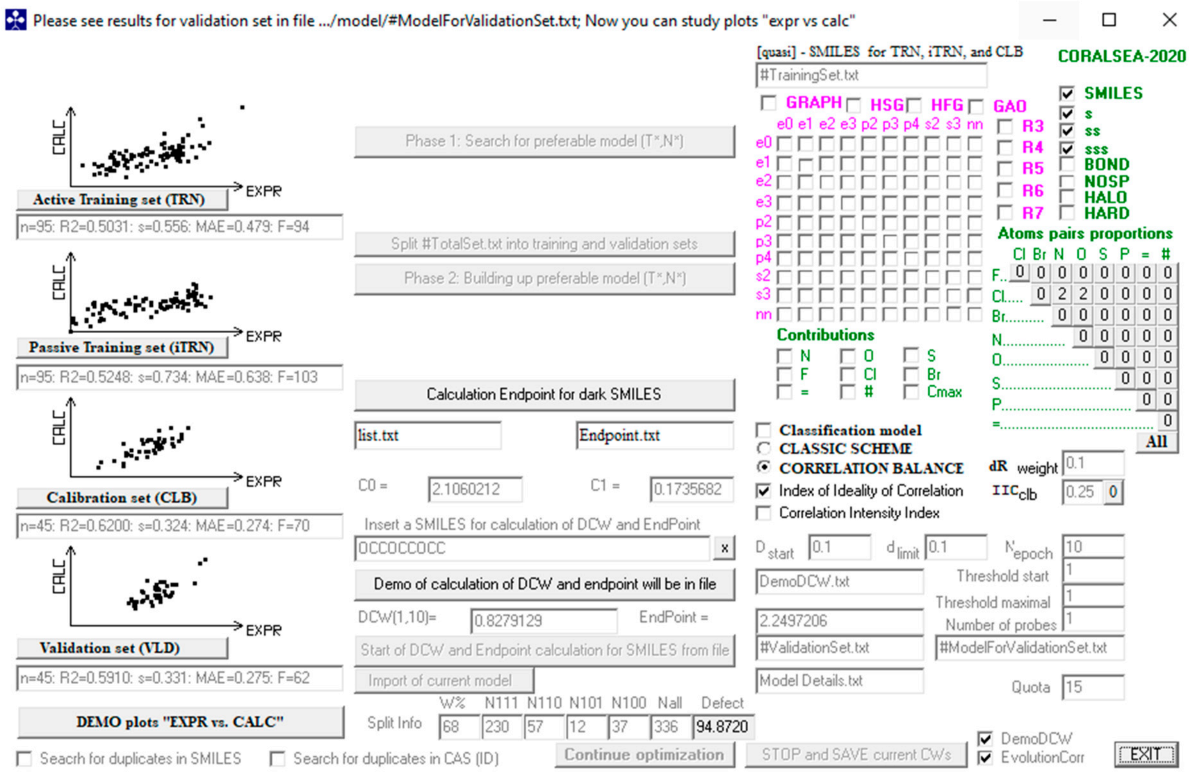

Figure S11. Screenshot of the method M3 utilized to build up the kidney NOAEL model in the CORAL software.

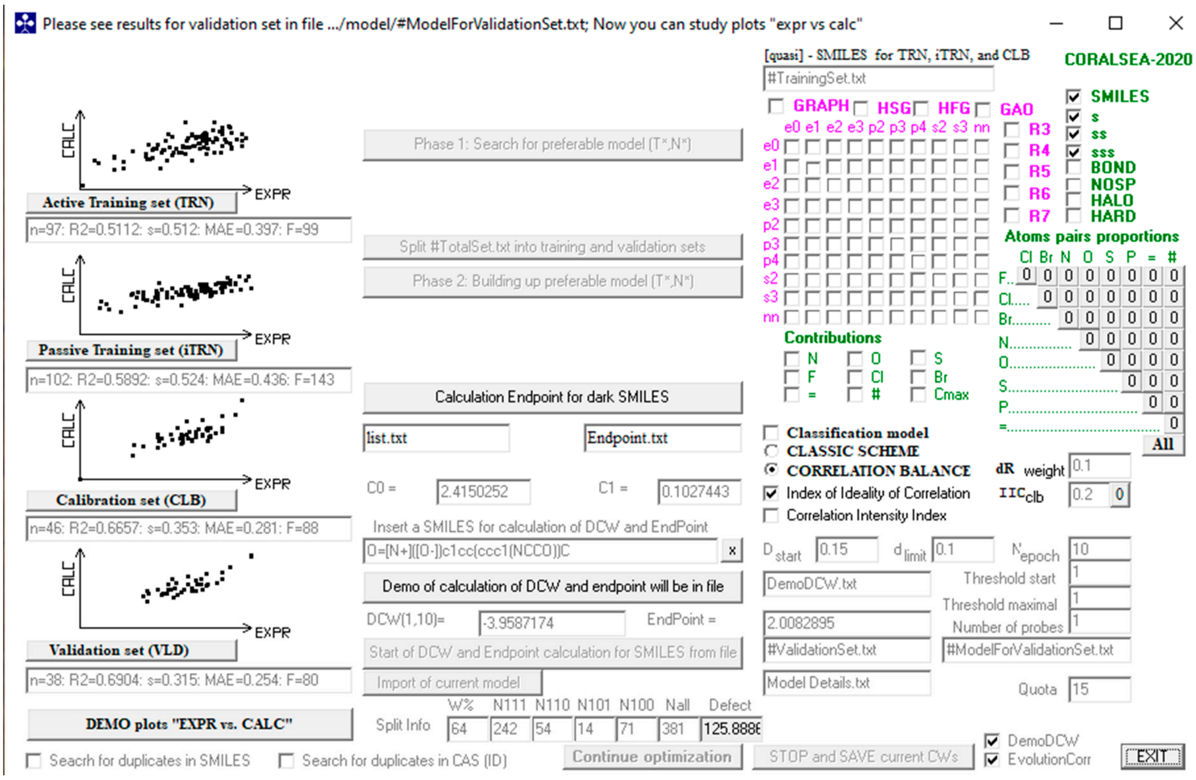

Figure S12. Screenshot of the method M4 utilized to build up the kidney LOAEL model in the CORAL software.

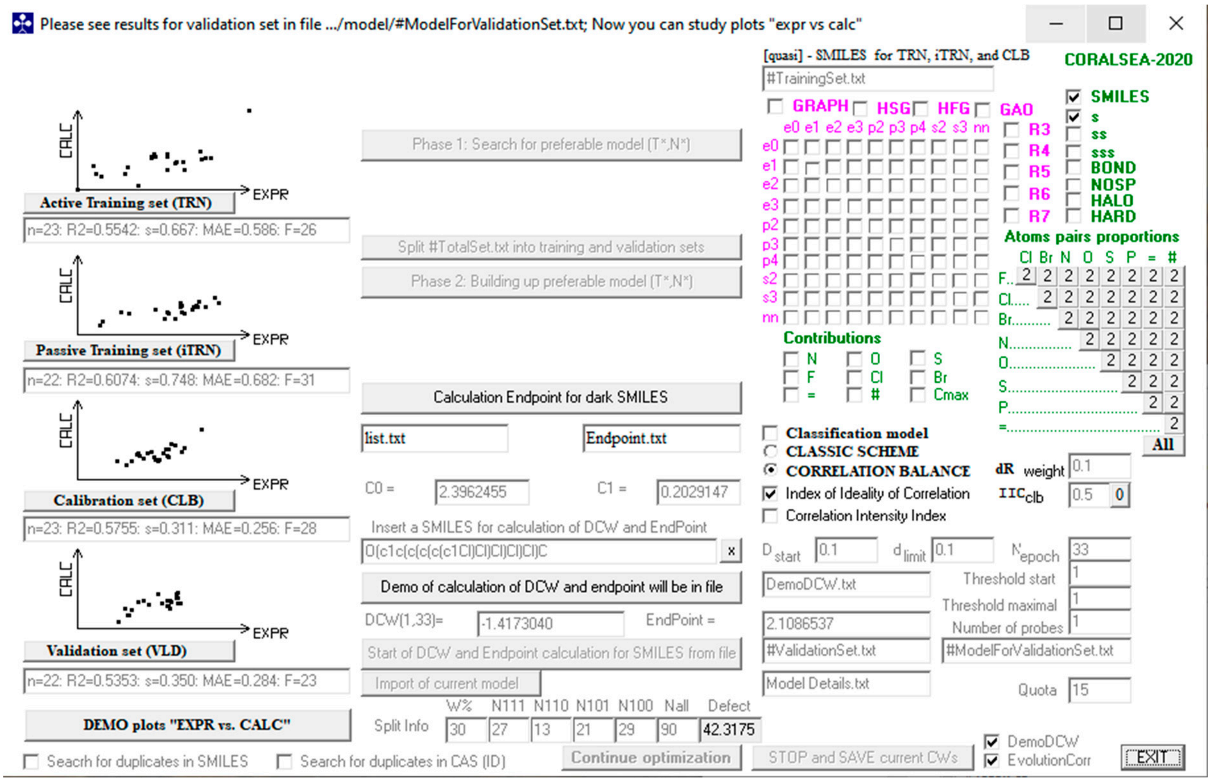

Figure S13. Screenshot of the method M5 utilized to build up the brain NOAEL model in the CORAL software.

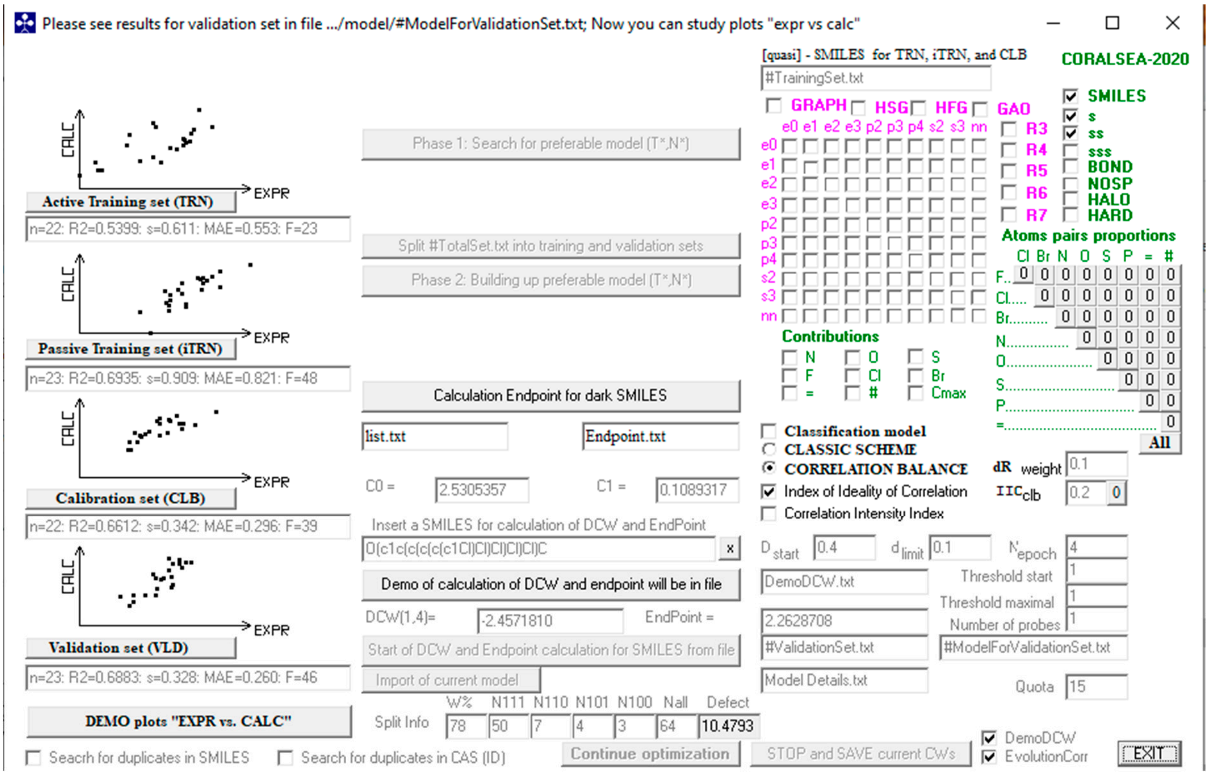

Figure S14. Screenshot of the method M6 utilized to build up the brain LOAEL model in the CORAL software.

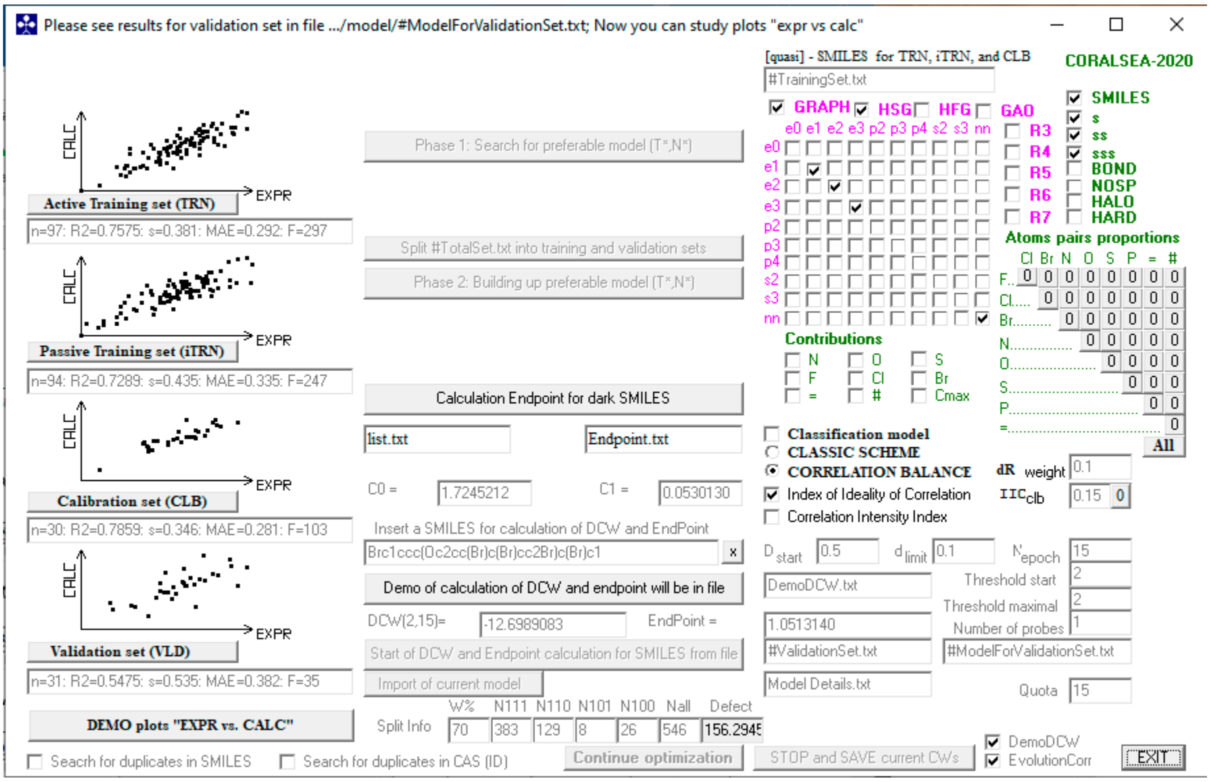

Figure S15. Screenshot of the method M7 utilized to build up the liver NOAEL model in the CORAL software.

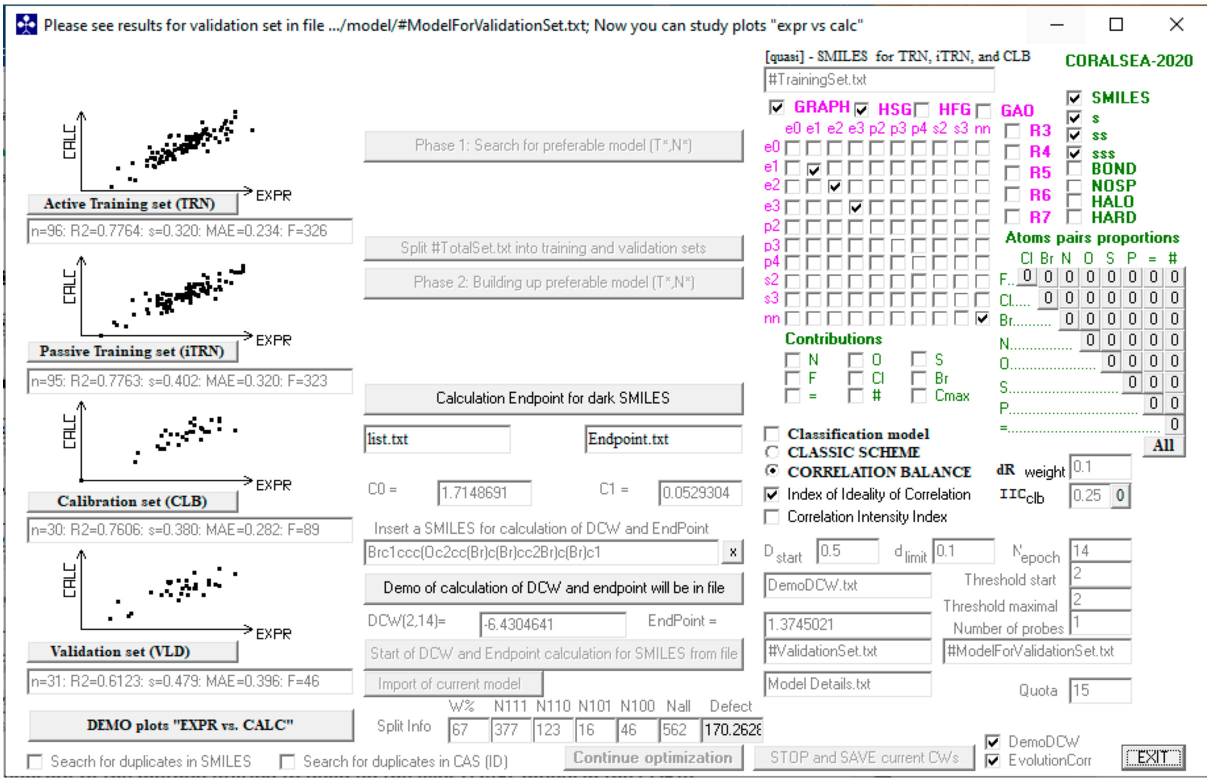

Figure S16.Screenshot of the method M8 utilized to build up the liver LOAEL model in the CORAL software.

Table S25. Molecular properties for the general dataset.

		Dataset – General		
		Mean	Maximum	Minimum
**Properties**	Mannhold logP	2.145	5.640	0.250
	Atomic polarizabilities	35.242	129.580	5.229
	Bond polarizabilities	19.926	94.926	1.680
	Hydrogen bond acceptors	2.684	14	0
	Hydrogen bond donors	0.977	9	0
	Rotatable bond count	7.110	28	0
	Lipinski’s Rule of Five	0.208	3	0
	Topological polar surface area	58.594	200.530	0
	Molecular weight	256.156	977.340	32.026

Table S16. Molecular properties for the kidney dataset.

		Dataset – Kidney		
		Mean	Maximum	Minimum
**Properties**	Mannhold logP	2.147	4.980	0.470
	Atomic polarizabilities	35.638	129.580	8.593
	Bond polarizabilities	19.785	94.926	2.520
	Hydrogen bond acceptors	2.740	11	0
	Hydrogen bond donors	0.862	5	0
	Rotatable bond count	7.076	23	0
	Lipinski’s Rule of Five	0.156	2	0
	Topological polar surface area	58.489	187.710	0
	Molecular weight	260.548	733.513	58.042

Table S27. Molecular properties for the brain dataset.

		**Dataset –Brain**		
		Mean	Maximum	Minimum
**Properties**	Mannhold logP	2.119	3.660	0.360
	Atomic polarizabilities	37.343	68.886	5.229
	Bond polarizabilities	20.808	45.481	2.520
	Hydrogen bond acceptors	3.000	11	0
	Hydrogen bond donors	0.769	3	0
	Rotatable bond count	8.099	23	1
	Lipinski’s Rule of Five	0.220	2	0
	Topological polar surface area	60.259	161.090	0
	Molecular weight	281.363	502.127	32.026

Table S28. Molecular properties for the liver dataset.

		Dataset –Liver		
		Mean	Maximum	Minimum
**Properties**	Mannhold logP	2.121	4.980	0.140
	Atomic polarizabilities	36.018	129.580	9.214
	Bond polarizabilities	20.134	94.926	2.520
	Hydrogen bond acceptors	2.677	14	0
	Hydrogen bond donors	0.790	5	0
	Rotatable bond count	7.459	28	0
	Lipinski’s Rule of Five	0.201	2	0
	Topological polar surface area	57.416	193.910	0
	Molecular weight	269.843	793.357	60.069
